# Is Sadness Only One Emotion? Psychological and Physiological Responses to Sadness Induced by Two Different Situations: “Loss of Someone” and “Failure to Achieve a Goal”

**DOI:** 10.3389/fpsyg.2017.00288

**Published:** 2017-03-03

**Authors:** Mariko Shirai, Naoto Suzuki

**Affiliations:** ^1^Graduate School of Psychology, Doshisha UniversityKyotanabe, Japan; ^2^Research Fellow of Japan Society for the Promotion of ScienceTokyo, Japan; ^3^Department of Psychology, Doshisha UniversityKyotanabe, Japan

**Keywords:** sadness, types of sadness, loss of someone, failure, physiological response

## Abstract

We investigated whether sadness elicited by two different situations—loss of someone (loss) and failure to achieve a goal (failure)—had different physiological responses. Seventy-four participants were randomly assigned to one of three conditions (loss, failure, and neutral). Physiological responses were recorded during an imagery task that was designed to evoke sadness. The results of characteristics in the subjective ratings indicated that loss-evoked sadness was only associated with expressive words relating to tears. For the results of physiological measures, skin conductance levels (SCLs) increased during the imagery task across all conditions and differed between conditions during the post-task. For the loss condition, restoration to baseline level took longer, while in the failure the SCL decreased sharply back to baseline. Furthermore, tear ratings correlated with blood pressures in the loss condition, while sadness intensity correlated with blood pressures in the failure condition. These results suggest that sadness includes at least two subtypes that produce different responses in subjective ratings and physiological measures.

## Introduction

The feeling of sadness is deeply ingrained in a person’s life. It occurs when one loses a valued person, fails to achieve a goal, or loses their sense of control (Ekman, 1999). Although various situations can cause sadness (Scherer and Wallbott, 1994), the question remains: are all of these feelings of sadness really the same? Several findings have shown that a single emotion can have different subtypes. For example, Nesse (1990) proposed that if different emotions corresponded to different kinds of situations, different subtypes of fear might develop to deal with different types of threats. Different expressions of anger: one with low pitch and one with high pitch, suggest that there are different types of anger associated with threat and frustration, respectively (Frick, 1986). Furthermore, Yoder et al. (2015) reported that physical disgust and moral disgust were separate emotions, which are each associated with different facial expressions. Considering these findings, it is possible that there are also different types of sadness.

In an earlier study Shirai and Suzuki (2015) examined the characteristics of sadness based on six situations via subjective ratings. The characteristics were assessed using expressive words for the following three factors: “tears,” “chest ache,” and “powerlessness.” Participants were asked to imagine the situation at four time points (event occurrence, after 1 week, after 1 month, and after 6 months), and assess the characteristics at each time point. The results showed that sadness induced by the loss of a family member (loss) tended to be expressed in words relating to “tears” and the ratings of the three factors remained high over time. In contrast, the features of sadness induced by the failure to achieve a goal (failure) decreased over time. According to the results, Shirai and Suzuki (2015) identified that sadness has at least two subtypes at the subjective level. Based on these findings, if sadness is regarded as an emotion with subtypes, the physiological responses attached to these subtypes could also differ.

Physiological responses to feelings of sadness have been examined in many studies to demonstrate the psychophysiological differences between emotions (e.g., Kreibig et al., 2007). Some studies have reported that when we feel sadness, heart rate (HR) accelerates or the skin conductance level (SCL) increases (e.g., Ekman et al., 1983), while others have reported that HR decreases or the SCL drops (e.g., Krumhansl, 1997). One potential reasons for these discrepant results relates to the use of different techniques to elicit emotion. However, Kreibig et al. (2007) argued that these inconsistent results could not be solely attributed to differences in the elicitation methods because both HR acceleration and deceleration have been seen with the same induction methods. Moreover, according to a meta-analysis of the existing studies on autonomic specificity, the exact nature of sadness is still quite vague in comparison to other emotions (Cacioppo et al., 2000).

Considering the previous inconsistent study results with regards to physiological responses, it is possible that different subtypes of sadness are associated with distinctive physiological responses. However, most previous studies have considered sadness to be one emotion rather than examining its different types. It is likely that different types of sadness have been treated as the same emotion in the research so far, and physiological responses to sadness have thus yield mixed results. It is important to clarify psychological and physiological response to sadness in order to understand the nature of sadness. Furthermore, if there are different types of sadness, then care should be taken to distinguish them from one another, which is believed to be worthwhile.

In terms of sadness subtypes, Shirai and Suzuki (in press) cite research examining physiological responses. They examined the physiological responses to the feelings of sadness elicited by loss and failure, with consideration of the different subjective types of sadness. The results showed that diastolic blood pressure (DBP) only increased during loss imagery but not in failure imagery. Such DBP increase in the loss condition can be interpreted as an increase in physical activity. In addition, the “tears” rating (one of the factors showing the characteristics of sadness) in the loss condition was higher than in the failure condition. Considering these DBP and tear rating changes, it may be suspected that sadness characteristics of tears reflect increased physical activation shown in DBP, as crying is an arousing behavior associated with physiological activation (Gross et al., 1994). However, there are two possible reasons for these differences. One is that they reflect the actual characteristics of the loss type of sadness; the other is that they are derived from the imagery task scripts because they included a description of crying over the loss of one’s grandfather. Crying implies that tears are shed, which would generate a high tear rating. To clarify the differences within what is presently the single category of “sadness,” we need to rectify these scripts and re-explore the physiological responses associated with the feelings of sadness.

Experimenters have been searching for a reliable method to elicit emotions when exploring emotional phenomena. It is difficult to elicit emotion in the laboratory because emotion itself is such an elusive construct, and no single technique can elicit all emotions equally (Rottenberg et al., 2007). Procedures that have been used in the past to induce emotions in the laboratory include: viewing images, sounds, films and imagined scenes (e.g., Lang, 1978; Rottenberg et al., 2007). Imagery, in particular, is a convenient method used to evoke emotional responses in a laboratory setting and facilitates psychophysiological measurement of emotional responses to the imagined events (Allen et al., 1996). Further, Cuthbert et al. (1991) argued that the physiological output is indicative of a person engaging in imagery as if it were the actual situation. Therefore, following the previous study (Shirai and Suzuki, in press), we decided to use this imagery technique to evoke sadness in the laboratory. To induce a strong intensity of emotion, participants were allowed to choose situations to imagine. Furthermore, Velasco and Bond (1998) pointed out the importance of personal relevance. They reported that more personally relevant emotional scripts have resulted in a more physiological arousal than non-personally relevant scripts have. Therefore, we targeted participants who had experienced events like those used in the emotional scripts before.

The aim of the present study was to examine whether physiological responses to sadness, as elicited by loss and failure, are different. To test this, we examined physiological responses to sadness elicited in an imagery task. For physiological measures, we focused on autonomic nervous system activity. In previous studies on physiological responses to sadness, HR, SCL, systolic blood pressure (SBP), and DBP have been the standard (Kreibig, 2010). Moreover, differences in DBP have also been shown in a previous study (Shirai and Suzuki, in press). Therefore, in the present study we measured physiological changes in relation to HR, SCL, SBP, and DBP. In addition, Kreibig (2010) suggested that “activating sadness” is associated with increased cardiovascular sympathetic control, whereas “deactivating sadness” is associated with sympathetic-parasympathetic withdrawal. Based on this suggestion, we targeted the high-frequency component of heart rate variability (HF) because, as an index, it reliably reflects parasympathetic activation (Pomeranz et al., 1985). In addition, physiological responses were measured after the imagery task. Physiological changes may manifest differently during the post-task because sadness has specific duration compared to other emotions. Scherer and Wallbott (1994) reported that sadness is a long-lasting emotion, which can often last for several days. Verduyn et al. (2011) also identified that sadness is prolonged compared to other emotions. In addition, studies showing different types of sadness at the subjective level have taken time course into consideration as a factor (Shirai and Suzuki, 2015).

In light of the above, we hypothesized that the psychological and physiological responses associated with loss and failure may differ. Based on previous findings, we predicted that sadness in the loss condition would be characterized by the tear rating, an increase of DBP during the imagery task, and long-duration of physiological responses. In contrast, in the failure condition, we predicted that there would be no changes in DBP, and that short-duration of physiological responses would be associated with sadness.

## Materials and Methods

### Participants

Participants were 74 (20 males, 54 females) undergraduates from Doshisha University, with a mean age of 19.82 years (*SD* = 1.41). Participants received 1000 yen (approximately USD 10) for their participation.

### Emotion Scripts

Six scripts (Supplement A in Supplementary Data Sheet 1) were created based on previous research (Shirai and Suzuki, 2013). These described situations that were designed to elicit emotions: two scripts each for the “loss” and “failure” types of sadness and two scripts for the “neutral” condition. Participants were randomly assigned to one of the three conditions (loss: *n* = 22, failure: *n* = 27, and neutral: *n* = 25). Before the imagery task, participants were asked to choose one of the two scripts. In the sadness conditions (loss or failure), participants chose the script that they felt elicited the greatest sadness for them, while in the neutral condition, they chose one of the two situations that elicited no emotion for them. In addition, all participants reported that they previously had a similar experience to the one described in the script.

### Imagery Task

The imagery task was designed with reference to Monchi and Suzuki (2000). It involved listening to descriptions of a certain situation recorded by the researcher and then imagining that situation. Participants were asked to listen to the recordings via headphones (MDR-2007, SONY).

After putting on the headphones, participants were instructed to keep their eyes closed and try to vividly visualize the content of the recording and imagine that it was really happening. The recordings for each situation were divided into three steps and were designed to induce sadness when the participant listened to the third step. After listening to the first step, participants imagined the situation and were then instructed to push a sensor button if they could clearly imagine the recorded situation. If they could not imagine it, they were instructed to not push the button. Participants performed the task at their own pace. After the researcher had confirmed that the button had been pushed, the next step of the recording was presented. This sequence was applied to all three steps. Subsequently, in the third step, the participants were asked to continue imagining the situation for 1 min (imagery period). Finally, they were asked to stop imagining the situation and wait for 5 min (post-period) before being instructed to open their eyes.

### Measures

#### Emotional Experience

To verify that the imagery task elicited the target emotional states, participants’ self-reported emotions experienced during the task were assessed with a comprehensive questionnaire comprised of the same scales used to rate their baseline state. The intensity of experiencing six emotions (fear, anger, disgust, happiness, anxiety, and sadness) was assessed using a 7-point Likert scale, ranging from (1) not at all to (7) very much, because these emotions tend to be experienced together with sadness (Power, 1999).

#### Characteristics of Sadness

A questionnaire was used to assess the characteristics of sadness (Shirai and Suzuki, 2015). It consisted of the three factors: “tears,” “chest ache,” and “powerlessness,” which were rated on a 7-point Likert scale that ranged from (1) not being expressed to (7) being expressed very well.

#### The Possibility of Coping with the Situation

The extent to which participants felt that they could cope with sadness was assessed using a Visual Analog Scale (VAS). The scale consisted of a 100 mm horizontal line, on which the participants indicated their level of coping with the situation by indicating their position on the line between the two end points. This was administrated to participants who reported feeling sad during the task as well as in their reports on the characteristics of sadness.

#### Imagery Task Evaluation

Participants rated how clearly they imagined the situation, using a 5-point Likert scale ranging from (1) not clear to (5) very clear. Further, the vividness of the situation was assessed using a 4-point Likert scale that ranged from (1) ambiguous to (4) very vivid. Finally, participants were asked if they had previously experienced a similar situation.

#### Physiological Measures

The physiological measures were continuously recorded from the start of the orientation period until the end of the post-period. Electrocardiograph (ECG) recordings were made using a one-lead configuration with three disposable Ag/AgCl electrodes (GE Healthcare Japan, SDC112) attached to each forearm. HR (in beats per minute) was analyzed using Acknowledge software that detects R-waves in the ECG and calculates each successive R-R interval. The maximum HR was set at 180 beats/min and the minimum at 40 beats/min.

HRV parameters were derived from the continuous ECG recordings by analyzing the variability in the R-R intervals, using the MemCalc/Tonam2C analysis program (Suwa Trust, Inc.). Frequency domain analyses were used: separated R – R intervals into frequency bands and then determined the power of each band during each period. Fast Fourier transform analyses were used to transform R-R intervals into high-frequency bands (HF: 0.15–0.40Hz). HF (ms^2^) is considered to be a specific marker of parasympathetic nervous system activity.

Skin conductance level (in microsiemens) was recorded using a constant-voltage device maintained at 0.5 V (Vega Systems, DA3-b), with two disposable electrodes (TEAC, PPS-EDA) attached to the index and ring fingers of the participant’s left hand. The data were amplified using DC amplifiers.

Systolic blood pressure and DBP (in millimeters of mercury) were measured continuously with MUB101 (Medicence, Inc.), a continuous blood pressure monitor, through a finger cuff attached to the middle finger of the participant’s left hand.

All signals except HF were sampled by a digital converter system (MP100, BIOPAC Systems) set at 500Hz and recorded on to a computer (Dell Optiolex 960).

### Procedure

First, the participants signed a consent form informing them of the physiological measurement and sadness-inducing imagery task requirements. After agreeing to take part, the cuff and electrodes were attached, and the participants were asked to find a comfortable sitting position and to try not to move as much as possible.

Participants completed a 5-min orientation period to accustom them to the laboratory setting and to check that whether the equipment was functioning as intended. Next, they completed a 3-min baseline recording of physiological activity, after which they were asked to fill out questionnaires that assessed their emotional state before the task (baseline state). Following this, the participants completed the practice task and were asked if they understood the task requirements. The experimenter confirmed that they understood the requirements and initiated the imagery task. After completing the task, participants provided ratings of the emotional state, vividness, and clarity of the visualization. In addition, when the participants reported feeling sad during the task, they were asked to rate the characteristics of their feeling of sadness and the possibility of coping with the situation. After completing the subjective ratings, the sensors and electrodes were removed, and participants were debriefed. A short, funny movie that was unrelated to the research was presented to help them recover from the induced state of sadness. The total experiment time was 50 min. This procedure was approved by the ethics committee of Doshisha University (15013).

### Data Analysis

The two scripts for each condition were averaged for all variables and the analyses were conducted using SPSS for Windows, Version 22.

#### Emotional Experience

The extent to which participants experienced each emotion during the imagery task were defined as change scores for each emotion, calculated by subtracting the baseline scores from the scores during the imagery task. These change scores were analyzed using two-way repeated measures analysis of variance (ANOVA) with emotion and condition as independent factors. For all other subjective rating measures, raw ratings were analyzed. Imagery task evaluation data were analyzed using one-way ANOVA. In addition, paired *t*-tests were used to analyze the scores for the sadness characteristics.

#### Physiological Measures

Some data were removed due to equipment failure or being ±2*SD* over the mean value during baseline. Continuous recordings of psychological responses were analyzed using Psychophysiological Analysis software (AcqKnowledge ver. 3.8.2, Biopac Systems). HF was averaged for every 5 s and recorded using MemCalc/Tonam2C.

Prior to the statistical analyses, the mean values of all physiological data were calculated for the last minute of a 3-min baseline period, and for 1 min of the imagery period (image) and post-period, respectively. The post-period was averaged per minute (p1–p5). All analyses were conducted using the mean change scores. The method used to calculate the mean change score is outlined below. The physiological measures from the baseline period were first tested for significant univariate differences between conditions. After no differences at baseline were confirmed (Supplement B in Supplementary Data Sheet 1), data for each period were transformed into change scores by subtracting the baseline mean from the mean of each period. Physiological mean change scores were statistically analyzed using a two-way repeated-measures ANOVA with period and condition as the independent factors. The Greenhouse-Geisser correction factor, *𝜀*, was used if the sphericity assumption was violated. Significant ANOVA results were followed-up with *post hoc* analyses using Tukey’s HSD tests to examine which data combinations differed significantly.

## Results

### Psychological Measures

#### Emotional Experience

**FIGURE 1:**
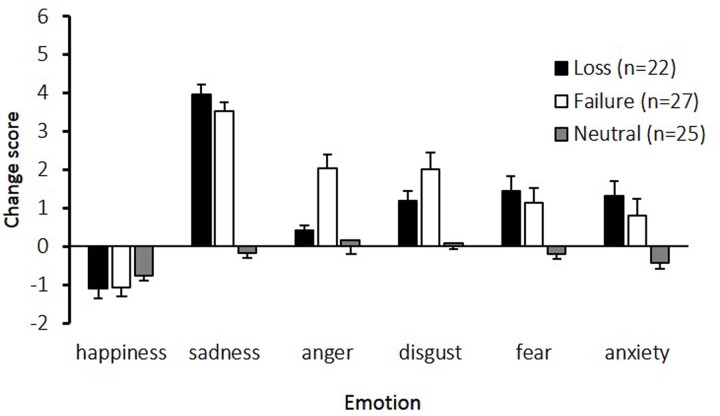
**Change scores for the six emotions experienced under the three conditions.** The horizontal axis shows the six emotions and the vertical axis shows the change scores. Participants assessed their subjective feelings of six emotions at baseline and after an imagery task. The change scores, calculated by subtracting the baseline scores from the scores in imagery task, are presented. The error bars represent standard error.

**Figure 1** shows the change in emotional state while participants performed the imagery task. The main effects of emotion [*F*(5,355) = 46.58, *p* < 0.01, *𝜀* = 0.84, *η*^2^ = 0.34], and condition [*F*(2,71) = 30.25, *p* < 0.01, *η*^2^ = 0.46], and the interaction between emotion and condition [*F*(10,355) = 10.29, *p* < 0.01, *𝜀* = 0.84, *η^2^* = 0.15] were significant. *Post hoc* comparisons indicated that the sadness change scores in the loss and failure conditions were the highest among the six assessed emotions. Furthermore, compared to the neutral condition, all emotions except happiness were significantly higher in the loss and failure conditions. The change scores for anger were higher in the failure condition than in the loss and neutral conditions.

#### Characteristics of Sadness

**FIGURE 2:**
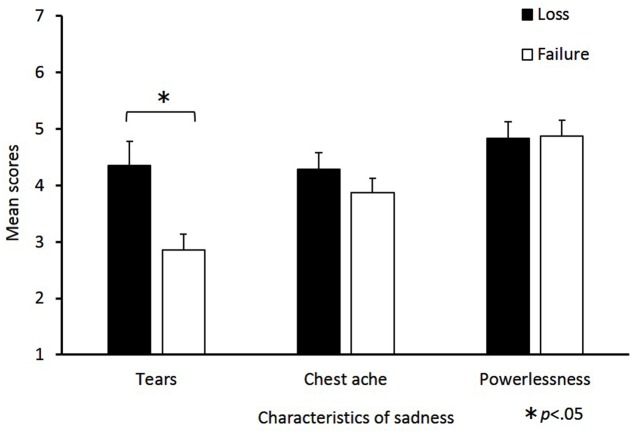
**Mean scores for sadness characteristics in the loss and failure conditions.** The horizontal axis shows the factors of the sadness characteristics and the vertical axis shows the mean change scores. Sadness characteristics consist of the following three factors: “tears,” “chest ache,” and “powerlessness.” Mean scores were calculated for each factor, the error bars indicate standard error. Significant differences are indicated by ^∗^*p* < 0.05.

The mean scores of sadness characteristics for the loss and failure conditions are shown in **Figure 2**. Independent-samples *t*-tests revealed that the tear value in the loss condition was significantly greater than in the failure condition [*t*(47) = 3.01, *p* = 0.01, *d* = 0.87]. By contrast, the differences between the loss and failure conditions in chest ache [*t*(47) = 1.07, *p* = 0.29, *d* = 0.31] and powerlessness [*t*(47) = 0.11, *p* = 0.92, *d* = 0.03] were not significant.

#### The Possibility of Coping with Sadness and Imagery Task Evaluations

**Table 1** shows the mean ratings from the imagery task evaluation and the possibility of coping with sadness. An independent-samples *t*-test evaluating the possibility of coping with sadness revealed that the difference between the loss and failure conditions was not significant. There was, however, a marginal trend toward significance [*t*(47) = 1.84, *p* = 0.07, *d* = 0.53], whereby ratings in the failure condition tended to be higher than in the loss condition. A one-way ANOVA found no significant main effects of clarity [*F*(2,71) = 0.92, *p* = 0.40, *η*^2^ = 0.03], or vividness [*F*(2,71) = 0.39, *p* = 0.77, *η*^2^ = 0.01].

**Table 1:** Mean (standard error) ratings on the imagery task evaluation and the possibility of coping with sadness for the three conditions.

Condition	Imagery task evaluations		The possibility of coping with the situation
	Clarity	Vividness	
Loss	3.73 (0.18)	2.82 (0.17)	3.23 (0.52)
Failure	3.59 (0.14)	2.74 (0.13)	4.62 (0.53)
Neutral	3.88 (0.13)	2.92 (0.14)	

### Physiological Measures

#### Skin Conductance Level

**FIGURE 3:**
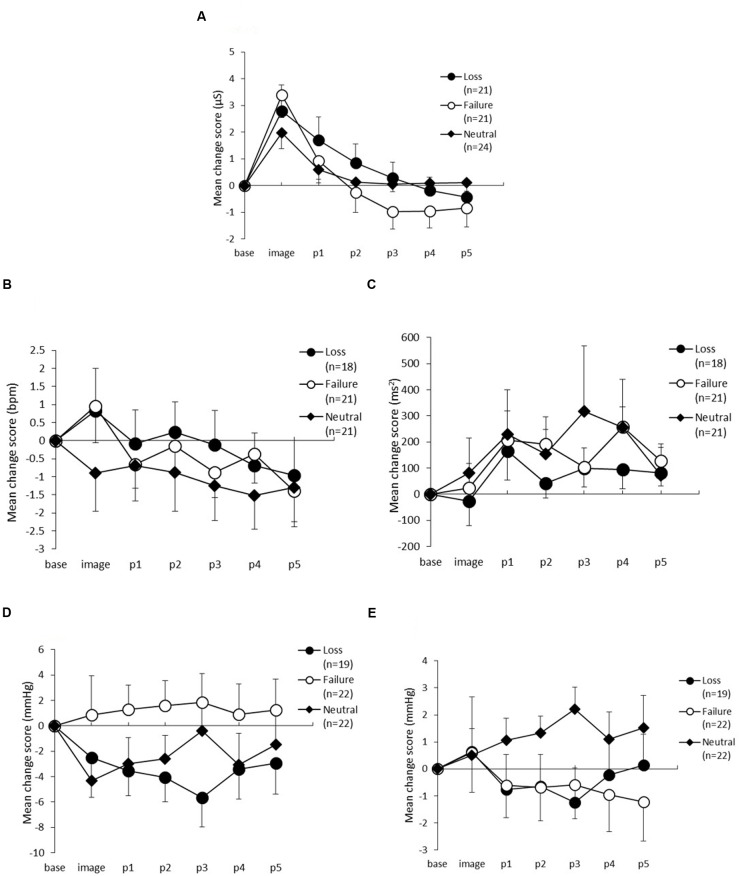
**Change scores from baseline to the post-period in (A)** Skin conductance level, **(B)** Heart rate, **(C)** High-frequency component of heart rate variability, **(D)** Systolic blood pressure, and **(E)** Diastolic blood pressure. The horizontal axis shows the time course (image, and p1–p5) and the vertical axis shows the variation in each measure. The error bars indicate standard error.

Skin conductance level changes between the three conditions are shown in **Figure 3A**. The ANOVA yielded a significant main effect of period [*F*(5,315) = 46.37, *p* < 0.01, *𝜀* = 0.35, *η*^2^= 0.40], and an interaction between period and condition [*F*(10,315) = 3.29, *p* < 0.01, *𝜀* = 0.35, *η*^2^= 0.06], but no significant main effect of condition [*F*(2,63) = 0.31, *p* = 0.73, *η*^2^= 0.01]. *Post hoc* comparisons showed that the SCL in the loss condition during p2 was significantly lower than during the imagery task (*α* = 0.05). Furthermore, the SCL in the failure condition during p1was significantly lower than during the imagery task (*α* = 0.05). The SCL during p2 was not significantly lower than during p1; however, this difference was marginally significant (*α* = 0.10). In the neutral condition, the SCL during p1 was significantly lower than during the imagery task (*α* = 0.05), but there were no differences in the other periods.

#### Heart Rate and Heart Rate Variability

**Figures 3B,C** show the changes in HR and HF. For HR, there were no statistically significant main effects of condition [*F*(2,57) = 0.35, *p* = 0.70, *η*^2^= 0.01] nor an interaction between period and condition [*F*(10,285) = 0.69, *p* = 0.67, *𝜀* = 0.73, *η*^2^= 0.02], but the main effect of period was statistically significant [*F*(5,285) = 3.49, *p* = 0.01, *𝜀* = 0.73, *η*^2^= 0.06]. *Post hoc* analyses did not identify any differences between the three conditions.

For HF, which is one of the indices of HRV and is thought to reflect parasympathetic activity, there were no statistically significant main effects for either condition [*F*(2,57) = 0.25, *p* = 0.77, *η^2^* = 0.01] or period [*F*(5,285) = 2.19, *p* = 0.09, *𝜀* = 0.62, *η*^2^= 0.04] and there was no interaction between period and condition [*F*(10,285) = 0.52, *p = 0*.80, *𝜀* = 0.62, *η*^2^= 0.02].

#### Systolic Blood Pressure and Diastolic Blood Pressure

The changes in SBP are shown in **Figure 3D**. The ANOVA revealed no main effects of condition [*F*(2,60) = 1.63, *p* = 0.20, *η*^2^= 0.05], or period [*F*(5,300) = 0.16, *p* = 0.87, *𝜀* = 0.48, *η*^2^= 0.00] and no interaction between period and condition [*F*(10,300) = 0.67, *p* = 0.64, *𝜀* = 0.48, *η*^2^= 0.02]. **Figure 3E** shows the variation in DBP across the three conditions. The ANOVA demonstrated no significant main effects of condition [*F*(2,60) = 0.88, *p* = 0.42, *η^2^* = 0.03], or period [*F*(5,300) = 0.49, *p* = 0.64, *𝜀* = 0.46, *η^2^* = 0.01], and no interaction between condition and period [*F*(10,300) = 1.39, *p* = 0.27, *𝜀* = 0.46, *η*^2^= 0.04].

#### Correlation between Psychological and Physiological Measures

To assess the relationship between the psychological and physiological measures, Pearson correlation coefficients were calculated between the sadness ratings, the characteristic of sadness, and the physiological measures change scores in the two sadness conditions (**Table 2**). In the failure condition, a positive association were observed between the intensity of feeling sad and the change scores for systolic (*r* = 0.66) and DBP (*r* = 0.59). Moreover, in the loss condition, positive associations were observed between the tear ratings and the change scores for both systolic (*r* = 0.63) and DBP (*r* = 0.59).

**Table 2:** Correlations between the psychological and physiological measures in the loss and failure conditions.

		SCL	HR	HF	SBP	DBP
	Sadness	-0.23	-0.03	0.10	0.29	0.18
Loss	Tears	0.26	0.45	-0.06	0.63^∗^	0.59^∗^
	Chest ache	-0.06	-0.18	0.23	0.07	0.11
	Powerlessness	0.08	0.33	-0.19	0.10	0.00
	Sadness	0.23	0.05	-0.07	0.66^∗^	0.59^∗^
Failure	Tears	0.19	0.15	0.06	0.24	0.32
	Chest ache	0.03	0.05	0.11	0.12	0.20
	Powerlessness	-0.22	-0.06	0.27	0.35	0.20


^∗^*p* < 0.05.

#### Additional Analyses

To evaluate the effects of gender on sadness intensity and the imagery task evaluation, *t*-tests were conducted for each measure. The number of males and females in each condition were as follows: the loss condition (4 males, 18 females), the failure condition (11 males, 16 females), and the neutral condition (5 males, 20 females). Independent-samples *t*-tests on sadness intensity revealed that male and female ratings did not differ significantly in either the loss condition (male: *M* = 3.00, *SE* = 0.41; female: *M* = 4.17, *SE* = 0.29; *t*(20) = 1.77, *p* = 0.09), or in the failure condition (male: *M* = 3.91, *SE* = 0.42; female: *M* = 3.25, *SE* = 0.27; *t*(25) = 1.40, *p* = 0.17).

Independent-samples *t*-tests were conducted to evaluate the clarity and vividness of the imagery task. For clarity, ratings by males and females did not differ significantly in the loss condition (male: *M* = 4.00, *SE* = 0.00; female: *M* = 3.67, *SE* = 0.23; *t*(20) = 0.67, *p* = 0.51), in the failure condition (male: *M* = 3.54, *SE* = 0.25; female: *M* = 3.62, *SE* = 0.18; *t*(25) = 1.05, *p* = 0.79), or in the neutral condition (male: *M* = 3.60, *SE* = 0.24; female: *M* = 3.95, *SE* = 0.15; *t*(23) = 1.05, *p* = 0.30). For vividness, ratings by males and females did not differ significantly in the loss condition (male: *M* = 2.75, *SE* = 0.25; female: *M* = 2.83, *SE* = 0.20; *t*(20) = 0.19, *p* = 0.86), in the failure condition (male: *M* = 2.91, *SE* = 0.25; female: *M* = 2.63, *SE* = 0.15; *t*(25) = 1.02, *p* = 0.32), or in the neutral condition (male: *M* = 2.60, *SE* = 0.24; female: *M* = 3.00, *SE* = 0.16; *t*(23) = 1.15, *p* = 0.26).

## Discussion

The main aim of the current study was to investigate whether there were differences in the physiological patterns related to feelings of sadness elicited from two different situations: loss and failure. For psychological measures, the change scores for sadness in the loss and failure conditions were the highest of the six emotions. The change scores for anger were higher in the failure condition than in the loss and neutral conditions. Regarding the characteristics of sadness, tear ratings were significantly higher in the loss condition than in the failure condition. For physiological measures, SCL changed along the time course differently between the two sadness conditions.

### Emotional Experience and the Characteristics of Sadness

With regards to emotional state, the findings demonstrated that the imagery in the two situations evoked sadness, with participants in the loss and failure conditions reporting more sadness compared to other emotions. In addition, the sadness change scores did not differ significantly between both sadness conditions; thus, there was no significant difference in the intensity between the loss and failure conditions. By contrast, the change in anger ratings in the failure condition was higher than in the loss condition. Ellsworth and Tong (2006) reported that self-anger — when one blames oneself— is usually combined with sadness, and guilt or embarrassment. It is believed that self-anger is evoked in the context of failure, as failure to attain the goal indicates a lack of self-ability.

More importantly, analysis of the characteristics of sadness indicated that the tear rating was higher in the loss condition than in the failure condition. Tears are a social signal to ask for help (Hendriks et al., 2007). The higher tear ratings in the loss condition are believed to be due to the features of a loss situation being more passive, and there is a smaller possibility of changing the results by oneself compared to a failure situation (Shirai and Suzuki, 2013). This finding is consistent with previous findings (Shirai and Suzuki, 2015, in press) and suggests that the sadness states in loss and failure reflect subjectively different types of sadness.

### Physiological Responses to the Imagery Task

An SCL increase during the imagery task was shown to occur across the three conditions. This indicates that performing the imagery task elicited activation of the sympathetic nervous system, consistent with the findings of Shirai and Suzuki (in press). Moreover, post-task physiological responses differed between the three conditions.

In the neutral condition, the SCL was significantly lower during p1 in the imagery period, and a significant change relative to p1 was not observed thereafter. Thus, these findings indicated that activation resulting from the imagery task sharply decreased back to baseline. For the failure condition, the change was similar to that in the neutral condition. The SCL significantly decreased relative to the imagery period at p1, however, a marginally significant difference between p1 and p2 was observed. In the loss condition, a significant difference was found between the imagery period and p2; thus, in terms of time to restore to the baseline level, more time was taken to recover in the loss condition than in the failure condition. These results imply that the physiological responses associated with the sadness elicited by loss differ from those associated with the sadness elicited by failure.

One reasons for the present findings may be the characteristics of the situations. In the present study, the findings for the possibility of coping score did not show a clear difference, but the raw score was high in the failure condition compared to the loss condition. In addition, Shirai and Suzuki (2013) reported that there was a greater possibility of changing the outcomes by oneself in failure situations compared to loss situations. Thus it is believed that in a situation like the loss of a loved one, we cannot deal with the event and feel passive. This feature influences the duration of the tension and sadness characteristics as time passes (Shirai and Suzuki, 2015). Furthermore, Sander and Scherer (2009) identified that the appraisal process of a situation affects the intensity and duration of sadness, which maintains anxiety and produces the possibility of depression. Therefore, such a trait could be responsible for the longer time taken to restore the baseline SCL. On the other hand, a failure situation presents greater possibility for coping compared to loss because one can find an alternative goal, unlike in the context of the loss of a loved one (Shirai and Suzuki, 2015). In failure situations coping is active rather than passive. This feature might cause a sharp decline in tension and sadness characteristics after a situation occurs as was shown by Shirai and Suzuki (2015). In light of these considerations, it is possible that the sharp decrease in SCL after the task was affected by the characteristics of the situation.

Apart from SCL, the other physiological measures did not change during the imagery task in all situations. Many studies have reported no change in HR as a result of sadness induction (e.g., Allen et al., 1996; Shirai and Suzuki, in press), while other studies have shown that SBP and DBP increase during sadness (Prkachin et al., 1999). In addition, our own previous research has shown that DBP increases only in the loss condition (Shirai and Suzuki, in press) and the results of the present study are inconsistent with this. There are some factors indicating that there are inconsistencies in the results of previous research. One should consider the script: in the present study, we changed the scripts to focus on situations because the previous scripts included a specific behavior, such as crying. The increase in DBP shown in our previous study (Shirai and Suzuki, in press) may not be due to different types of sadness but to the script itself. However, as psychological ratings were almost consistent with the findings of our earlier research, we cannot definitely conclude why there were inconsistencies in the physiological responses.

Regarding the DBP increase in the loss condition, in our previous work we identified the possibility that the tear rating increased with DBP (Shirai and Suzuki, in press). To clarify the relationship between the psychological and physiological measures, we examined the correlation between these measures under both loss and failure conditions. The results showed that there were positive associations between the tear ratings and the change scores for SBP and DBP in the loss condition. In contrast, positive associations were observed between the sadness intensity ratings and the SBP and DBP change scores in the failure condition. From these results, it can be concluded that different ratings were associated with the changes in blood pressure in each sadness condition. The results in the loss condition are in line with our earlier work (Shirai and Suzuki, in press), suggesting that the DBP increase in the loss condition might be due to the tear rating. Therefore, although blood pressure did not change during the task under both sadness conditions, these results suggest that blood pressure changes might be influenced by different factors in each condition.

The above results suggest that the physiological responses attached to loss and failure differ. Therefore, it is possible that different types of sadness exist in terms of the psychological and physiological responses. Gender effects were also considered as an alternative factor that might influence responses, other than the subtype of sadness. For example, Vingerhoets and Scheirs (2000) reported that women cry more frequently and intensely than men. With regards to sadness intensity, we compared males and females in each condition but the results indicated no significant differences. Furthermore, on the clarity and vividness of imagery, there were also no significant differences. Thus, gender differences are unlikely to substantially influence our findings.

We should acknowledge several limitations in this study. First, we did not account for individual differences; whereas research has shown that the relationship between imagery ability and physiological activity are related to individual differences in imagery ability (Miller et al., 1987). Screening participants with good imagery ability would be required to control for this. A second limitation was that we targeted participants who had previously experienced actual events like those described in the emotional scripts, to evoke intense sadness. However, most of the physiological responses did not change during the task, potentially due to the weak intensity of emotion. In future, an alternative method or task would be required to elicit more intense and pure sadness, such as recalling autobiographical situations (Marci et al., 2007). Finally, we focused only on physiological responses. Moors (2009) stated that emotions need to be thought of from four perspectives: cognitive, feeling, motivational and somatic, and action. In the present study, different types of sadness were considered from the subjective and physiological perspectives since our results suggest that the physiological and psychological responses to sadness that are elicited from these two situations do differ. In order to demonstrate the existence of a subtype of sadness more clearly, further research on alternative measures, such as the elements of facial expressions, would be desirable.

## Conclusion

We found that during an imagery task, the physiological responses to sadness elicited from two different situations did not differ substantially, but they did show clear differences during the post-task period. Furthermore, changes in blood pressure were influenced by different factors in each sadness condition. For psychological response, loss-evoked sadness was strongly related to tears. Therefore, the results suggest that different types of sadness exist in terms of psychological and physiological responses.

## Author Contributions

MS and NS conceived the experiment. MS conducted the experiment and analyzed the results. MS and NS interpreted the results. All authors reviewed the manuscript.

## Conflict of Interest Statement

The authors declare that the research was conducted in the absence of any commercial or financial relationships that could be construed as a potential conflict of interest.
