# Supplementary material for: Is Sadness Only One Emotion? Psychological and Physiological Responses to Sadness Induced by Two Different Situations: “Loss of Someone” and “Failure to Achieve a Goal”
Source: Front Psychol. 2017 Mar 3;8:288. doi: 10.3389/fpsyg.2017.00288 (PMC5334320; doi:10.3389/fpsyg.2017.00288)
Supplement: Supplementary file 1 [file Data_Sheet_1.pdf]

Supplement A - Six emotional scripts

Loss (1. death of your grandfather)

1. While you are at university, a family member calls you as your grandfather has fallen ill and been taken to hospital. Please imagine the situation of receiving this call.
2. You hang up and your heart skips a beat. Please imagine the scenario of you deciding to go to the hospital immediately.
3. When you arrive at the hospital, your grandfather has already died. Please imagine you saw grandfather's face passed away.

Loss (2. death of your pet)

1. Your pet dog becomes ill. You devote a lot of attention to your pet. Please imagine your pet is very weak.
2. Please imagine feeding your pet as usual as you think this is best.
3. Later, your pet seems asleep, you touch and realize it is dead. Please imagine that you see your pet.

Failure (1. failure to pass the university entrance examination)

1. You have studied extremely hard to pass you entrance exam during high school days. Please imagine the today is the day that results come out.
2. You are in front of the results board which show the name of a person who passed. Please imagine you try to find your name on the board.
3. Please imagine you cannot find your name on the board.

Failure (2. failure to win a final club tournament)

1. You have trained very hard at your club during high school days. Today is the final match.

Please imagine yourself at the beginning of the final match.

2. Please imagine your match performance is below your practice standards.
3. Finally, please imagine you lose the final match.

Neutral (1. on the way to the university as a part of normal day)

1. Please imagine you are preparing to leave for school and walk out your door.
2. Please imagine you are walking to school and the traffic light is green.
3. Please imagine you walk towards the light, it changes to red, so you need to wait.

Neutral (2. preparing for the day after getting up in the morning)

1. Please imagine you are asleep and your alarm clock rings.
2. Please imagine going into bath room to wash your face and looking in a mirror.
3. You have washed and dried your face by the towel. Please imagine you are brushing your teeth.

Supplement B - No difference during baseline for five measures.

SCL (Loss: M = 2.70, Failure: M = 3.81, Neutral: M = 1.97, F (2, 63) = 2.91, *n.s.*, η^2 = .08), HR (Loss: M = 69.71, Failure: M = 69.05, Neutral: M = 69.61, F (2, 57) = .04, *n.s.*, η^2 = .00), HF (Loss: M = 509.67, Failure: M = 798.60, Neutral: M = 919.67, F (2, 57) = 2.00, *n.s.*, η^2 = .07), SBP (Loss: M = 130.84, Failure: M = 130.66, Neutral: M = 132.14, F (2, 60) = .09, *n.s.*, η^2 = .00), DBP (Loss: M = 65.67, Failure: M = 69.33, Neutral: M = 65.92, F (2, 60) = 1.61, *n.s.*, η^2 = .04).
